# Cognitive Effects of Astaxanthin Pretreatment on Recovery From Traumatic Brain Injury

**DOI:** 10.3389/fneur.2020.00999

**Published:** 2020-10-15

**Authors:** Chen Fleischmann, Esther Shohami, Victoria Trembovler, Yuval Heled, Michal Horowitz

**Affiliations:** ^1^The Institute of Military Physiology, IDF Medical Corps, Tel-Hashomer, Israel; ^2^Heller Institute of Medical Research, Sheba Medical Center, Ramat Gan, Israel; ^3^Laboratory of Environmental Physiology, Hebrew University, Jerusalem, Israel; ^4^Department of Pharmacology, Institute for Drug Research, Hebrew University, Jerusalem, Israel; ^5^Kibbutzim College, Tel Aviv, Israel

**Keywords:** astaxanthin, traumatic brain injury (TBI), cognitive tests, neurological severity score (NSS), Y maze, object recognition test (ORT), nutritional supplementation Astaxanthin pretreatment and TBI recovery

## Abstract

Traumatic brain injury (TBI), caused by mechanical impact to the brain, is a leading cause of death and disability among young adults, with slow and often incomplete recovery. Preemptive treatment strategies may increase the injury resilience of high-risk populations such as soldiers and athletes. In this work, the xanthophyll carotenoid Astaxanthin was examined as a potential nutritional preconditioning method in mice (sabra strain) to increase their resilience prior to TBI in a closed head injury (CHI) model. The effect of Astaxanthin pretreatment on heat shock protein (HSP) dynamics and functional outcome after CHI was explored by gavage or free eating (in pellet form) for 2 weeks before CHI. Assessment of neuromotor function by the neurological severity score (NSS) revealed significant improvement in the Astaxanthin gavage-treated group (100 mg/kg, ATX) during recovery compared to the gavage-treated olive oil group (OIL), beginning at 24 h post-CHI and lasting throughout 28 days (*p* < 0.007). Astaxanthin pretreatment in pellet form produced a smaller improvement in NSS vs. posttreatment at 7 days post-CHI (*p* < 0.05). Cognitive and behavioral evaluation using the novel object recognition test (ORT) and the Y Maze test revealed an advantage for Astaxanthin administration via free eating vs. standard chow during recovery post-CHI (ORT at 3 days, *p* < 0.035; improvement in Y Maze score from 2 to 29 days, *p* < 0.02). HSP profile and anxiety (open field test) were not significantly affected by Astaxanthin. In conclusion, astaxanthin pretreatment may contribute to improved recovery post-TBI in mice and is influenced by the form of administration.

## Introduction

Traumatic brain injury (TBI), caused by mechanical impact to the brain, is a leading cause of death and disability among young adults (1). Soldiers (2) and athletes (3), exposed to rigorous conditions and combat hazards, face a high risk of sustaining TBI. Wartime TBI is often caused by blast or concussive injury: a non-penetrating closed head injury (CHI). Complications include sleep disorders (4), anxiety and depression (5), chronic pain (6), memory and other cognitive impairments (7), increased risk of posttraumatic stress disorder (8, 9), and behavioral (2), sensory (10, 11), and immune disruptions (12). The mechanical brain trauma causes the accumulation of harmful mediators, such as reactive oxygen species (ROS) (13, 14), cytokines, free fatty acids, and excitatory amino acids leading to widespread cell death, through a cytotoxic cascade, which is the driving force to ensuing damage, morbidity, and disability (15). Physiological and psychological stress existing at the time of injury have a profound effect on its outcome (16, 17).

The prophylactic approach to TBI treatment has been extensively explored by pre-treatment with mild stress to induce increased resilience to injury. Limited success was attained with ischemia (18), hypothermia (19), heat stress (20, 21), endotoxin exposure (22), hypoxia (23), and heat acclimation (24, 25). Other preemptive methods utilize hyperbaric oxygen (26, 27), subtoxic doses of chemicals such as 3-nitropropionic acid, and pharmaceuticals including erythromycin, kanamycin, acetylsalicylic acid, 2-deoxyglucose, N-methyl-D-aspartate (NMDA) receptor antagonists (28), low-dose NMDA (29, 30), and even ethanol (31). Their potential adverse effects for long-term prophylaxis in high-risk populations have led to a search for safer substances, particularly nutraceuticals such as omega 3 (32). Astaxanthin is a xanthophyll carotenoid prevalent in marine organisms, approved for human consumption as a safe food supplement (33, 34). Astaxanthin assists in maintaining the integrity of cellular membranes by preventing lipid peroxidation (35). Many health-related benefits have been attributed to Astaxanthin (36–46). Astaxanthin was shown to be effective as a preemptive treatment to different stressors such as exercise in rodents (47, 48), heat-related injury (49), and ischemia–reperfusion injuries (IRIs) (50–54). In a rat model (55), Astaxanthin was shown to penetrate the blood–brain barrier (BBB), to reach therapeutic concentrations, and to exert its antioxidant, anti-inflammatory, and neuroprotective effects (56, 57). In a human study, Astaxanthin was demonstrated to improve the cognitive function in the elderly (58). Brain IRI models in Astaxanthin-supplemented rats before (59, 60) or following (61) IRI showed significantly decreased neuronal damage and neurological deficit scores, accompanied by a dose-dependent increase in heat shock protein 32 (HSP32) and HSP70. Heat shock proteins (HSPs) are important markers in the cellular stress response (62) and have valuable functions in neuroinflammation and neuronal survival (63). Recently, Astaxanthin supplementation displayed cognitive benefits when administered to mice following a CHI model. Results displayed advantages for Astaxanthin treatment postinjury in the neurological severity score (NSS), Y Maze, and object recognition test (ORT) (64).

The aim of this study was 2-fold: (1) to investigate the effect of Astaxanthin pre-treatment on HSP dynamics and functional outcome after CHI and (2) to evaluate the effects of Astaxanthin preparations, gavage or free pellet eating, on motor and cognitive function, within 1 month of follow-up after CHI.

## Materials and Methods

### Ethics

Experimental procedures were approved by the authority for biological and biomedical models ethics committee for animal experimentation of the Hebrew University, Jerusalem, Israel (Approval numbers: MD-13-13734-4 and MD-16-14842-4) and complied with the guidelines of the national research council guide for the care and use of laboratory animals (65).

### Animals

Male sabra mice aged 5–7 weeks were included. Pre-experiment, all animals were housed in a controlled environment (12–12 h light–dark cycle and 24 ± 1°C). Food (Teklad Global rodent diet no. 2018SC, 18% protein Harlan Teklad, USA, by Envigo, Israel) and water were provided *ad libitum*. Animal body weight was recorded biweekly.

### Astaxanthin Preparation

Astaxanthin 1% v/v for gavage was prepared by dissolving Astaxanthin 10% oleoresin (Astapure®, Algatechnologies, Ktora, Israel) in extra virgin olive oil (acidity ≤ 0.8%, Negba olive press, Revivim, Israel) by a 1:10 ratio. Astaxanthin 1% w/w handmade pellets for free eating were prepared by grinding rodent food pellets (standard chow) to a powder, mixing in the Astaxanthin 10% oleoresin, at a 1:10 ratio (50 g of Astaxanthin 10% oleoresin, 500 g of animal food powder), and wetting with animal drinking water (350 ml). Pellets were prepared, dried, and frozen.

### Closed Head Injury

A modified weight drop model was used, based on the work of Chen et al. (66), used frequently in both mice (67–70) and rats (71, 72). Briefly, isoflurane-anesthetized animals underwent a midline longitudinal incision to expose the skull, followed by the use of a calibrated weight-drop device, which allowed a Teflon-tipped cone (2 mm diameter, 95 g), to fall over the exposed skull covering the left cerebral hemisphere 1–2 mm lateral to the midline in the mid-coronal plane, to induce a focal injury to the left hemisphere. Sham-treated mice were anesthetized and underwent skin incision, but no CHI was delivered. Following sham or CHI induction, mice were returned to their cages for recovery. Food and water were provided *ad libitum*.

### Experimental Layout

**Figure 1:**
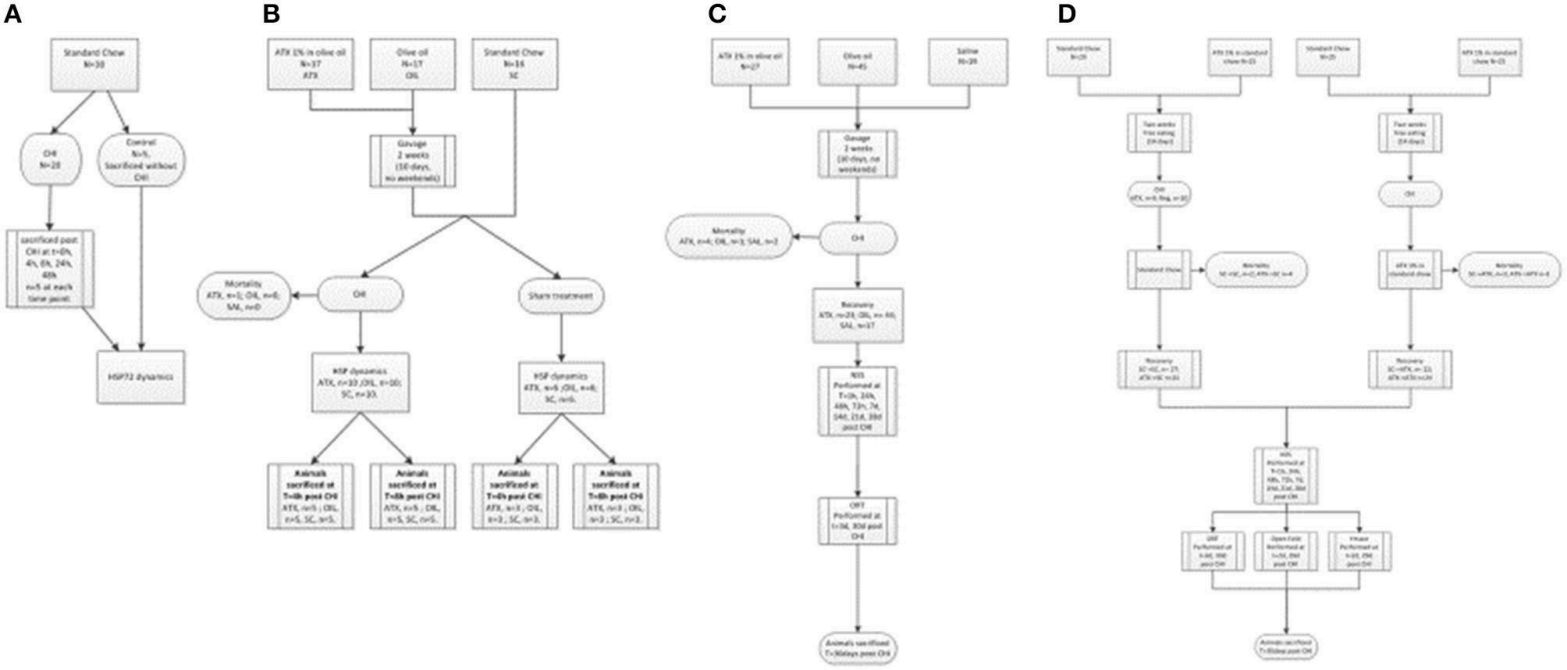
Flow-charts of experimental stages. **(A)** First stage (preliminary) – HSP72 dynamics post CHI (un-supplemented). **(B)** First stage – HSP dynamics post CHI (supplemented, by gavage). **(C)** Second stage – Gavage experiment, long term recovery. **(D)** Third stage - Pellet experiment, long term recovery.

The three experimental stages are depicted in Figure 1: the first stage was aimed to determine whether cellular protection by HSP72 immediately following CHI is affected by Astaxanthin pre-supplementation by gavage. The second stage examined neuromotor and short-term memory effects of Astaxanthin supplementation by gavage during 1 month of recovery. The third stage introduced Astaxanthin administration by free eating, and additional cognitive tests were performed within 1 month of recovery.

In the first stage, evaluation of left cortical HSP72 protein dynamics by Western immunoblotting was conducted at *t* = 0, 4, 8, 24, and 48 h in the left, injured, hemisphere of post-CHI or control mice (Figure 1A, *n* = 5/group). Next, the 4- and 8-h time points were chosen, and animals were administered either Astaxanthin 1% dissolved in olive oil at 100 mg/kg (ATX, *n* = 17) or olive oil (OIL, *n* = 17), at the same volume (0.1 ml/10 g body weight), by gavage for 2 weeks, excluding weekend (10 days) or standard chow (*n* = 16) for 2 weeks and then exposed to CHI or sham surgery (Figure 1B). Animals were sacrificed at 4 or 8 h post-CHI or sham and cortical HSP72, HSP27, HSP90, and HSF1 content in the affected left-brain hemisphere was assessed by Western immunoblotting. In the second stage, animals were administered either Astaxanthin 1% dissolved in olive oil at 100 mg/kg (ATX, *n* = 27), olive oil (OIL, *n* = 45), or normal saline (SAL, *n* = 19) at the same volume (0.1 ml/10 g body weight), by gavage for 2 weeks, excluding weekend (10 days), and then exposed to CHI (Figure 1C). Recovery consisted of 4 weeks of free eating and drinking, with follow-up assessments of the animals' motor function using the NSS (see below) performed at 1, 24, 48, and 72 h and at 7, 14, 21, and 28 days post-CHI, and short-term memory cognitive function using the novel ORT (see below) performed at 3 and 30 days post-injury. In the third stage, animals were administered either Astaxanthin 1% mixed in with ground standard chow presented in pellet form or standard chow by free eating in one of four combinations: 2 weeks of either standard chow (SCpre) or Astaxanthin 1% (ATXpre), followed by CHI and 1 month of recovery, during which the animals received either standard chow (SCpost) or Astaxanthin 1% in pellets (ATXpost), in the following group combinations: ATXpre_SCpost (*n* = 25), ATXpre_ATXpost (*n* = 25), SCpre_ATXpost (*n* = 25), SCpre_SCpost (*n* = 29) (Figure 1D). During the 4-week recovery period, the mice had free access to food and water, and their neurobehavioral function was assessed using the NSS, performed at 1, 24, 48, and 72 h and at 7, 14, 21, and 28 days post-CHI. Cognitive function was assessed by the ORT and Y Maze test performed at 3 and 30 and 2 and 29 days, respectively, post-injury. Anxiety level was assessed by open field (see below) and performed at 2 and 29 days post-injury.

### Behavioral Tests

#### Neurological Severity Score

The NSS for mice as previously described (66, 73–76) was used to evaluate the neuromotor status after CHI. The presence of reflexes and the ability to perform motor and behavioral tasks are scored from 0 to 10, increasing with the severity of dysfunction (73). The rate of recovery is represented by ΔNSS—the difference between the NSS at any time point and the initial injury severity (1 h post-CHI) (72). A trained technician, blinded to the treatment received by the animals, performed the test. NSS observations were made at 1, 24, 48, and 72 h and at 7, 14, 21, and 28 days post-CHI.

#### Object Recognition Test for Mice

Based on a test developed by Ennaceur and Delacour (77), and used extensively to assess short-term memory loss by rodents post-CHI (78–81), the ORT relies on the natural tendency of rodents to explore novelty. It was performed as described previously (82) with mild modifications, at 3 and 30 days post-CHI. Briefly, 24 h after a 10-min habituation to the testing environment (a square white Perspex arena of 50 × 50 × 30 cm), the mouse was subjected to the training stage, during which it was free to explore two identical objects for 5 min. Four hours later in the test stage, one object was replaced by a new, unfamiliar object, and the mouse was again allowed to explore the arena for 5 min. The tests were performed in a quiet room with low light. Cumulative time spent in object-directed exploratory behavior by the mouse at each object (touching, sniffing, scratching, and climbing) was recorded and scored. A preference toward the new unfamiliar object during the test stage is expected in healthy mice, whereas neurologically affected mice lack memory of the familiar object and spend an equal amount of time exploring both the new and familiar objects. The test exploration ratio was calculated as the exploration time of the new object divided by the exploration time dedicated to both objects. The greater this ratio value, the higher the recognition of novelty. The preference index (PI) was calculated as the difference between the exploration time dedicated to the old and new objects, divided by the exploration time dedicated to both objects. The change in exploration ratio (ΔER) represents the difference between the test and baseline part of the test for the changed object. Test results were compared between the treatments at 3 and 30 days post-CHI, during the baseline and test stages.

#### Y Maze

This spatial memory test was described previously (83–85). A maze built of three black Perspex arms at a 120° angle from one another (“start,” “other,” and “new” arms) is used for two separate attempts: first, the mouse is placed in the maze at the “start” arm and for 5 min, allowed to freely explore the “start” and “other” arms, while the “new” arm remains closed. Two minutes later, the mouse is returned to the maze and allowed to explore all three arms for 2 min. The amount of time spent in each arm is documented. Short-term memory of recognition is reflected by the novelty ratio (NR), calculated as the amount of time spent in the new arm relative to the total amount of time spent in the “other” plus “new” arms. The discrimination index (DI) is calculated as the difference between time spent in the “other” and “new” arms relative to the total amount of time spent in the “other” and “new” arms. The test was performed at 2 and 29 days post-CHI. Higher values of novelty recognition and of discrimination represent better performance of the spatial memory cognitive function.

#### Open Field

The open field test (86) is based on the behavioral tendency of mice in a heightened state of anxiety to refrain from exploring open unprotected areas and remain mainly at the periphery of the arena, whereas animals in a lower state of anxiety will tend to show a higher interest in freely exploring all areas of the arena. Activity level, as expressed by locomotor behavior, is also assessed in this test. The test was performed as previously described (87) with moderate alterations: a square white Perspex box was used as the arena (size, 50 × 50 × 30 cm). A smaller center zone was defined as 50% of the arena. The animal is introduced into the empty arena without previous acclimation and is left to freely explore the arena for 10 min with video monitoring from above. The arena is later analyzed as a periphery (50 × 50 cm) and a central area (50% in size). The test was performed at 2 and 29 days post-CHI. Motion in the arena was captured and analyzed using the Noldus “Ethovision” software, version XT 11, USA. Analyzed parameters included area preference (center vs. periphery), transition between areas, and locomotor activity.

#### Animal Sacrifice

The animals were sacrificed after the recovery period by sedation with isoflurane, followed by decapitation. Brain tissue was promptly removed, separated to the right and left cortex and hippocampus, placed in liquid nitrogen, and then moved to deep freezing at −80°C.

#### Western Immunoblotting

Left cortical tissue was prepared as described previously (88). Protein concentration was quantified by Bradford reagent (Bio-Rad, USA), and 50-μg samples were separated using 12% sodium dodecyl sulfate–polyacrylamide gel electrophoresis (SDS-PAGE) gel (TGX Fast cast, Bio-Rad, USA) and blotted onto nitrocellulose membranes, which were then blocked with 5% skim milk powder. Blots were probed overnight at 4°C with primary antibodies against HSP72, HSP27, HSP90, and HSF1. Anti-β-actin antibody was used to confirm equal protein loading. Appropriate peroxidase-coupled immunoglobulin G were used as secondary antibodies (1 h, at room temperature). Supplementary Table 1 lists the primary (Supplementary Table S1A) and secondary (Supplementary Table S1B) antibodies used. Membranes were stripped for reprobing when necessary by exposing them to guanidine thiocyanate 4 M solution for 30 s. Reactive bands were visualized using chemiluminescence (EZ-ECL, Biological Industries, Israel) and detected using the ChemiDoc imaging system (Bio-Rad, USA). Image lab software was used to measure band pixel density (version 5.1, Bio-Rad, USA), which was normalized to β-actin in the same lane. Four technical repeats were performed for each sample.

#### Statistical Analysis

Data are presented as mean ± standard error. The SPSS program (version 23, USA) was used for statistical analysis. Protein levels from immunoblotting were normalized to actin in the same lane. In the first stage of the experiment, independent samples *t-*tests were used to assess the significance of difference between average cortical HSP72 protein levels of mice sacrificed at each time point and the control group. In the following stage, left cortical levels of HSPs were analyzed for normality of distribution using the Kolmogorov–Smirnov test by protein, by the stress employed (TBI or sham treatment), and by time post-CHI (4 or 8 h) and then assessed for statistical significance between pre-treatment groups and stress conditions by one-way ANOVA followed by *post hoc* analysis with Tukey's test, for normally distributing parameters, or Kruskal–Wallis test followed by Mann–Whitney's U test for *post-hoc* analysis for non-normally distributing parameters. Motor and behavioral test results were tested for normality of distribution by the Kolmogorov–Smirnov or Shapiro–Wilks test and evaluated for significance using one-way ANOVA followed by Tukey's *post-hoc* analysis, or Mann–Whitney test for normally and non-normally distributing parameters, respectively. A *p* < 0.05 was considered significant for all comparisons.

## Results

No significant difference was observed in the change in body weight between experimental groups in each of the experimental stages (see Supplementary Table S2 for comparison of animal body weights in the free eating stage).

### First Stage

#### HSP Dynamics

**Figure 2:**
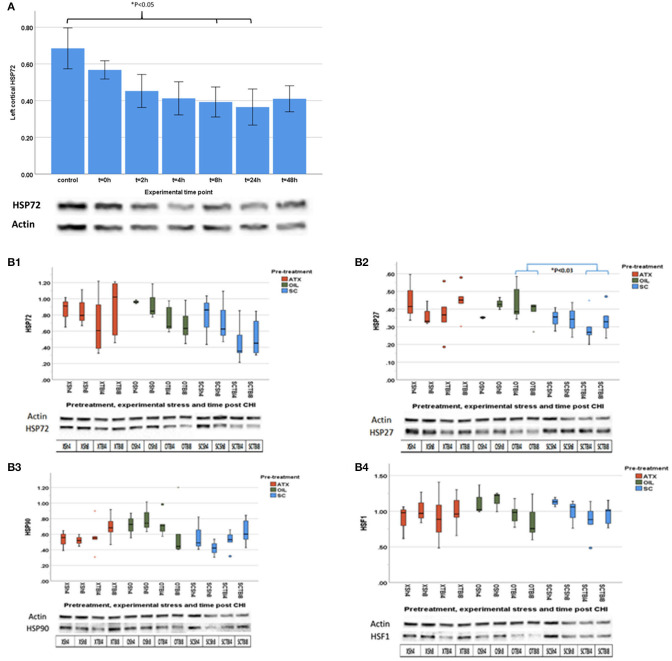
First stage results. **(A)** HSP72 dynamics post CHI (un-supplemented): Cortical HSP72 level post-CHI is shown for the affected left hemisphere. *t* = hours post-TBI; *n* = 4 mice per group, four repeats. Values normalized to pool, Student's *t*-test, one tailed, equal variance. A significant reduction from control values was observed beginning from *t* = 8 h until *t* = 48 h (*p* < 0.05). **(B)** HSP dynamics post CHI (supplemented). Left cortical levels of HSP's at 4 and 8 h post-CHI. Dark orange – ATX (X) group; Olive green – OIL (O) group; Blue – No treatment (SC) group. TBI – Traumatic brain injury group (*n* = 5–6 mice in each group); Sh – Sham group (*n* = 3–4 mice in each group); 4 repeats; 4 = 4 h post-CHI; 8 = 8 h post-CHI. Outliers: dot – Regular outliers, asterisk – extreme outliers. Bottom – sample of western blot membrane image. **(B1)** HSP72; **(B2)** HSP27; **(B3)** HSP90; **(B4)** HSF1.

Figure 2A demonstrates that CHI leads to a gradual reduction in cortical HSP72 protein level compared to the control in the affected brain hemisphere, which becomes significantly lower 8 h post-CHI and continues until 24 h post-CHI (Figure 2A, *p* < 0.05). Therefore, the 4- and 8-h time points post-CHI were chosen to evaluate the effect of Astaxanthin supplementation by gavage (ATX) on cortical HSPs (Figures 2B1–B4). Pretreatment with ATX displayed a non-significant trend toward recovery of cortical HSP72, while an opposite trend was observed in the OIL-treated group (non-significant). Analysis of HSP27 levels revealed a similar dynamic to that of HSP72 protein levels in the ATX-supplemented group (not statistically significant). In the OIL-treated group HSP27 protein level at 4 and 8 h was significantly higher than that of the un-supplemented group (SCTBI) (*p* < 0.03). No significant difference between groups was observed in HSP90 or HSF1 levels.

### Second Stage

#### NSS

**Figure 3:**
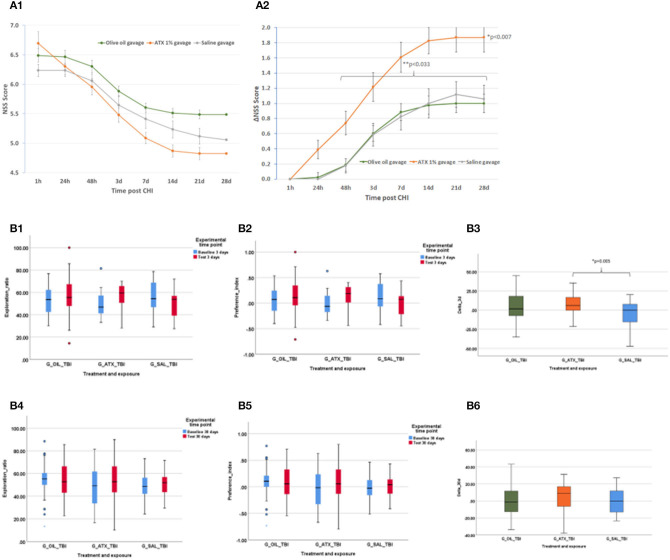
Second stage results. **(A)** NSS, stage 2 (gavage). NSS and ΔNSS values for stage 2 from 1 h to 28 days post CHI, ATX: orange (n = 23); OIL: green (n = 43); SAL – gray (n = 17). **(A1)** NSS values. **(A2)** ΔNSS. *P < 0.007 between ATX and OIL ΔNSS, at all time points; **P < 0.033 between ATX and SAL, from 48 h to the end of follow-up (28 d) (Mann-Whitney *U*-test). **(B)** ORT, stage 2 (gavage). Results of the ORT, gavage experiment at 3- and 30-days post-CHI; TBI, Traumatic brain injury; In Exploration ratio (ER) and Preference index (PI) graphs, baseline values appear in blue, test values appear in red. In ΔER graphs, ATX: orange (*n* = 23); OIL: green (*n* = 44); SAL: blue (*n* = 18); **(B1)** Exploration ratio (ER), 3 days. **(B2)** Preference index (PI), 3 days. **(B3)** ΔER (baseline to test), 3 days. At 3 days post-CHI, ΔER of the ATX group is significantly higher than that of the SAL group (*p* < 0.005, one-way ANOVA, Tukey *post-hoc* analysis). **(B4)** Exploration ratio (ER), 30 days. **(B5)** Preference index (PI), 30 days. **(B6)** ΔER (baseline to test), 30 days.

Initial NSS scores (1 h post-CHI) from all the experimental groups indicated moderate brain trauma (6.24 ± 0.11, 6.49 ± 0.11, 6.70 ± 0.19 for the SAL, OIL, and ATX groups, respectively) (Figure 3A1). ΔNSS during recovery in the ATX gavage group (ATX) was found to be significantly higher than that of the OIL gavage group (OIL) at all tested time points post CHI (*p* < 0.007, Figure 3A2) and higher than the saline gavage group (SAL) from 48 h post-CHI until the end of recovery (*p* < 0.033, Figure 3A2).

#### ORT

Figure 3B depicts the exploration ratio (ER) and preference index (PI) at 3 and 30 days, as well as the change (delta, Δ) in ER from baseline to test at days 3 and 30 post-CHI. At 3 days post-CHI, ΔER was significantly higher in the ATX group compared to the SAL group but not significantly higher than that of the OIL group (*p* = 0.005, Figure 3B3). No improvement in ER, PI, or ΔER was observed with either ATX or OIL supplementation compared to the SAL group at 30 days post-CHI (Figures 3B4–B6).

### Third Stage

#### NSS

**Figure 4:**
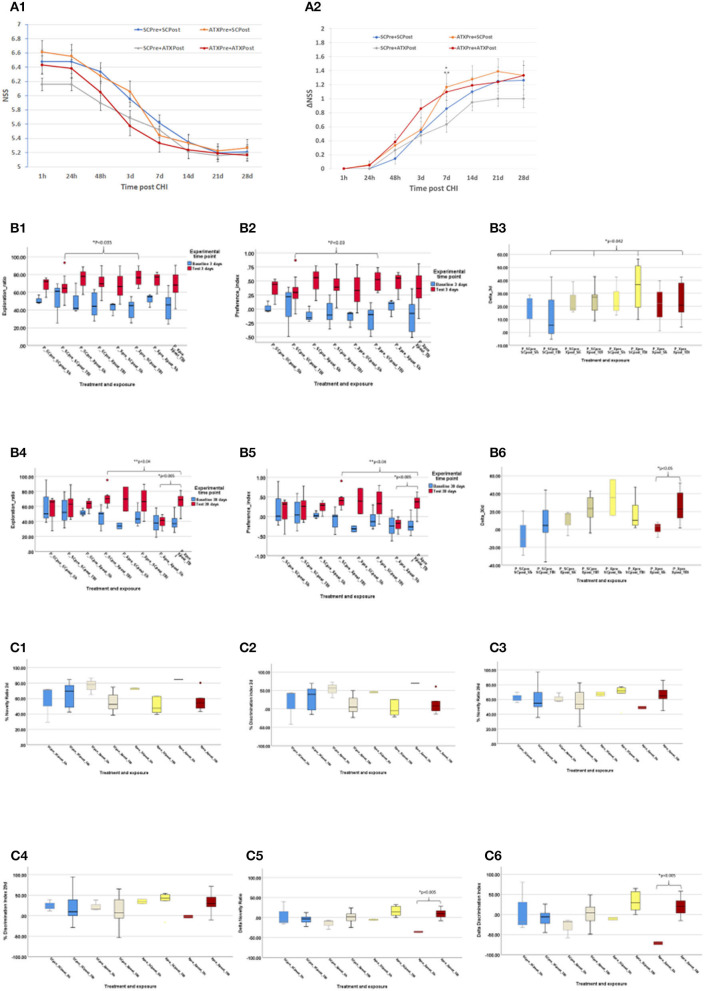
Third stage results. **(A)** NSS, stage 3 (pellets). NSS and ΔNSS values for stage 3 from 1 h to 28 days post-CHI, *n* = 15–17 per group; ATXPre+ATXPost – red; ATXPre+SCPost – orange; SCPre+ATXPost – gray; SCPre+ SCPost – blue; **(A1)** NSS values. **(A2)** ΔNSS. *P < 0.035 between ΔNSS of ATXPre+SCPost and SCPre+ATXPost; **P < 0.05 between ΔNSS of ATXPre+ATXPost and SCPre+ATXPost. (Mann-Whitney *U*-test). **(B)** ORT, stage 3 (pellets). Results of the ORT, pellet experiment at 3- and 30-days post CHI; TBI – Traumatic brain injury group (*n* = 10–12 per group); Sh, Sham experiment; (*n* = 3 per group). In Exploration ratio (ER) and Preference index (PI) graphs, baseline values appear in blue, test values appear in red. In ΔER graphs, ATXPre+ATXPost – dark red; ATXPre+SCPost – yellow; SCPre+ATXPost – beige; SCPre+ SCPost – blue; **(B1)** Exploration ratio (ER), 3 days. **(B2)** Preference index (PI), 3 days. **(B3)** ΔER (baseline to test), 3 days. At 3 days post-CHI, The ER and PI test values of P_Xpre_SCpost_TBI were significantly higher than those of P_SCpre_SCpost_TBI (*p* < 0.035, *p* < 0.03, respectively, Mann-Whitney *U*-test), and ΔER of the P_SCpre_SCpost_TBI group was significantly lower than all other TBI exposed treatment groups (*p* < 0.042, Mann-Whitney *U*-test). **(B4)** Exploration ratio (ER), 30 days. **(B5)** Preference index (PI), 30 days. **(B6)** ΔER (baseline to test), 30 days. At 30 days post-CHI, in both the ER and PI graphs, in the P_Xpre_Xpost group, the test score of the TBI exposed mice was significantly higher than that of the Sham treated mice (*p* = 0.005, independent samples *t*-test, 2-tailed, equal variance) Additionally, The ER and PI of the P_SCpre_Xpost_TBI were significantly higher than those of the P_Xpre_Xpost_TBI group (*p* < 0.04, one-way ANOVA, Tukey *post-hoc* analysis). At 30 days post-CHI, In the P_Xpre_Xpost group, the ΔER test score of the TBI exposed mice was significantly higher than that of the Sham treated mice (*p* < 0.05, independent samples *t*-test, 2-tailed, equal variance). **(C)** Y Maze: Results of the Y Maze, pellet experiment at 3- and 30-days post-CHI; TBI, Traumatic brain injury group; ATXPre+ATXPost – dark red (*n* = 9); ATXPre+RegPost – yellow (*n* = 5); RegPre+ATXPost – beige (*n* = 8); RegPre+ RegPost – blue (*n* = 9); Sh – Sham experiment (*n* = 2–3 per group). **(C1)** Novelty ratio (NR), 2 days. **(C2)** Discrimination index (DI), 2 days. **(C3)** Novelty ratio (NR), 29 days. **(C4)** Discrimination index (DI), 29 days. **(C5)** ΔNR, from 2 to 29 days. **(C6)** ΔDI, from 2 to 29 days. At 29 days post-CHI, the ΔNR and ΔDI of the TBI exposed ATXPre+ATXPost treatment group, group were significantly higher than those of the Sham exposed mice in the same treatment group (*p* < 0.005, one-way ANOVA, Tukey *post-hoc* analysis).

Figure 4A depicts the NSS score (Figure 4A1) and changes over time (ΔNSS, Figure 4A2) in the third stage. Initial NSS scores (1 h post-CHI) from all the experimental groups indicated moderate brain trauma (6.42 ± 0.14). Astaxanthin in pellet form, both as pre-treatment (ATXpre_SCpost) and the combined pre- and post-CHI treatment (ATXpre_ATXpost), displayed a significantly higher ΔNSS at 7 days, compared to post-CHI supplementation of Astaxanthin in pellet form (SCpre_ATXpost, *p* < 0.05 and *p* < 0.035, respectively, Figure 4A2).

#### ORT

Results of the third stage ORT are presented in Figure 4B, including ER, PI, and ΔER at 3 and 30 days post-CHI. At 3 days post-CHI, the ER and PI test values of P_Xpre_SCpost_TBI were significantly higher than those of P_SCpre_SCpost_TBI (*p* < 0.035 and *p* < 0.03, respectively). Moreover, all pellet forms of Astaxanthin administration displayed a significantly higher change in recognition rate from baseline to test (ΔER) than that of the control un-supplemented group (P_SCpre_SCpost_TBI, *p* < 0.042). At 30 days post-CHI, in both the ER and PI graphs, in the P_Xpre_Xpost group, the test score of the TBI-exposed mice was significantly higher than that of the sham-treated mice (*p* = 0.005). Additionally, the ER and PI of the P_SCpre_Xpost_TBI were significantly higher than those of the P_Xpre_Xpost_TBI group (*p* < 0.04), and a significantly higher ΔER test score was recorded for the TBI-exposed mice in the P_Xpre_Xpost group compared to the sham-treated mice in the same group (*p* < 0.05).

#### Y Maze

The NR and DI at 2 and 29 days post-CHI, as well as the change (Δ) in NR and in DI from 2 to 29 days post-CHI are depicted in Figure 4C. On day 29 post-CHI, 14 mice escaped from the maze: 2 sham-treated mice (ATXpre_ATXpost, *n* = 1; ATXpre_SCpost, *n* = 1) and 12 from the group exposed to CHI (ATXpre_SCpost, *n* = 5; ATXpre_ATXpost, *n* = 3; SCpre_ATXpost, *n* = 2; SCpre_SCpost, *n* = 2). They were removed from statistical analysis. At 29 days post-CHI, the ΔNR and ΔDI of the TBI-exposed ATXPre + ATXPost treatment group were significantly higher than those of the sham-exposed mice in the same treatment group (*p* < 0.005 each). No significant differences were observed between TBI-exposed mice of the different treatment groups at 2 or 29 days.

#### Open Field

Table 1 lists activity measures in the arena. Individual parameters are depicted in Supplementary Figure S2. No significant difference was observed between treatment groups and stress exposures on day 2 or 29 post-CHI. No effect on anxiety was observed.

**Table 1:** Open field results (Third stage).

**Parameter**	**Day post CHI**	**SC pre_SC post**				**SC pre_ATX1% post**				**ATX 1% pre_SC post**				**ATX1% pre_ATX1% post**			
		**TBI**		**Sham**		**TBI**		**Sham**		**TBI**		**Sham**		**TBI**		**Sham**	
		***N***	**Mean ± St.Dev.**	***N***	**Mean ± St.Dev.**	***N***	**Mean ± St.Dev.**	***N***	**Mean ± St.Dev.**	***N***	**Mean ± St.Dev.**	***N***	**Mean ± St.Dev.**	***N***	**Mean ± St.Dev.**	***N***	**Mean ± St.Dev.**
Distance moved (cm)	2	11	4357.73 ± 1214.79	3	4525.3 ± 1085.66	9	6028.96 ± 2214.35	4	5087.71 ± 494.34	9	4040.71 ± 862.97	4	4977.78 ± 1207.74	10	5176.86 ± 1413.37	4	4959.4 ± 1506.3
	29	12	4198.16 ± 1908.46	3	6111.96 ± 1890.83	8	4403.29 ± 426.65	4	4415.03 ± 1115.11	11	3677.94 ± 936.23	1		12	4760.68 ± 2530.15	3	4935.74 ± 1916.34
Velocity (cm/s)	2	11	7.26 ± 2.02	3	7.54 ± 1.8	9	10.07 ± 3.67	4	8.5 ± 0.81	9	6.74 ± 1.42	4	8.29 ± 2	10	8.65 ± 2.36	4	8.33 ± 2.53
	29	12	7 ± 3.17	3	10.2 ± 3.15	8	7.34 ± 0.7	4	7.37 ± 1.86	11	6.13 ± 1.56	1		12	7.93 ± 4.21	3	8.23 ± 3.2
Time in center (s)	2	11	69.27 ± 39.48	3	63.77 ± 38.05	9	66.56 ± 26.27	4	68.14 ± 11.2	9	54.87 ± 19.81	4	53.26 ± 7.96	10	74.13 ± 40.42	4	78.14 ± 53.34
	29	12	42.51 ± 21.87	3	52.68 ± 22.99	8	64.83 ± 26.69	4	43.53 ± 35.62	11	45.64 ± 25.06	1		12	53.89 ± 23.25	3	42.24 ± 28.8
Time in Periphery (s)	2	11	530.4 ± 38.96	3	536.21 ± 38.24	9	532.01 ± 24.84	4	530.16 ± 9.01	9	544.49 ± 19.92	4	546.76 ± 8.04	10	525 ± 39.69	4	520.97 ± 52.98
	29	12	556.62 ± 20.86	3	546.39 ± 21.88	8	535.06 ± 26.64	4	555.61 ± 37.1	11	503.06 ± 168.68	1		12	546.17 ± 23.21	3	557.28 ± 29.24
Activity in arena	2	11	0.74 ± 0.21	3	0.79 ± 0.08	9	0.95 ± 0.24	4	0.86 ± 0.21	9	0.74 ± 0.2	4	0.8 ± 0.15	10	0.84 ± 0.32	4	0.84 ± 0.32
	29	12	0.7 ± 0.23	3	1.03 ± 0.07	8	0.76 ± 0.16	4	0.84 ± 0.21	11	0.69 ± 0.23	1		12	0.74 ± 0.26	3	0.89 ± 0.41
Highly active duration	2	11	0.16 ± 0.28	3	0.31 ± 0.53	9	0.11 ± 0.23	4	0.23 ± 0.46	9	0.34 ± 0.56	4	0.15 ± 0.3	10	0.08 ± 0.25	4	0.13 ± 0.26
	29	12	0 ± 0	3	0 ± 0	8	0 ± 0	4	0 ± 0	11	0.05 ± 0.18	1		12	0 ± 0	3	0 ± 0
Moderately active duration	2	11	19.86 ± 24.38	3	15.71 ± 12.04	9	48.22 ± 50.2	4	33.38 ± 36.65	9	19.68 ± 29.11	4	27.54 ± 19.74	10	33.21 ± 52.87	4	21.31 ± 19.73
	29	12	22.24 ± 23.12	3	49.13 ± 31.43	8	21.84 ± 19.46	4	33.01 ± 27.33	11	19.71 ± 20.1	1		12	18.27 ± 20.48	3	58.64 ± 54.77
Inactive duration	2	11	561.56 ± 37.97	3	569.43 ± 22.65	9	521.17 ± 65.38	4	542.81 ± 54.82	9	565.8 ± 40.43	4	551.76 ± 32.64	10	545.28 ± 68.45	4	548.94 ± 45.27
	29	12	563.3 ± 35.46	3	515.19 ± 27.29	8	558.24 ± 32.73	4	541.77 ± 38.68	11	561.95 ± 35.43	1		12	559.08 ± 39.74	3	510.26 ± 77.3

*SC, Standard Chow; ATX1%, Astaxanthin in pellet form; TBI, exposure to traumatic brain injury; Sham, exposure to sham treatment; pre, before exposure; post, after exposure. N is listed in the table. No significant difference was found between groups (ANOVA)*.

## Discussion and Conclusions

**Figure 5:**
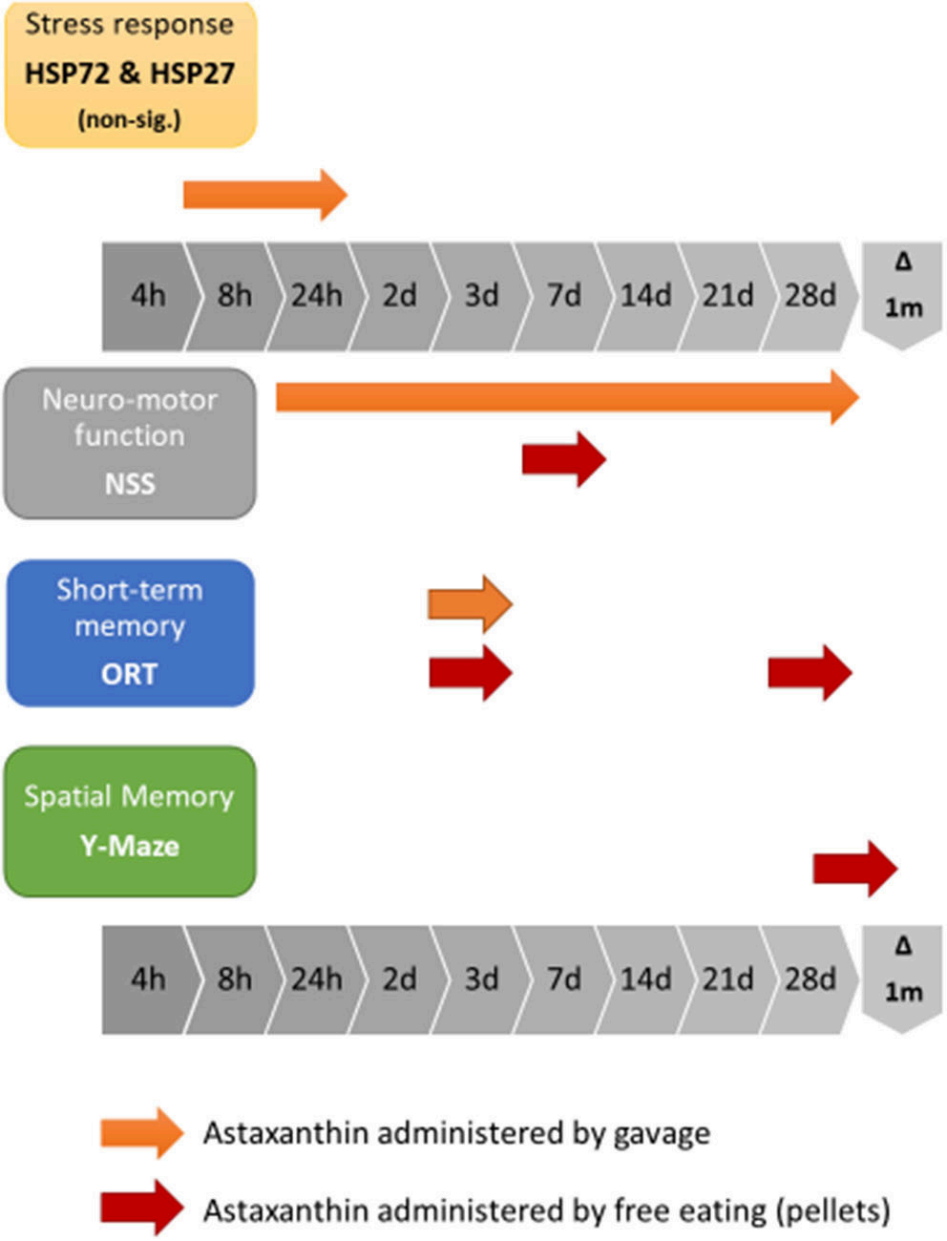
Graphic representation of the combined effects of Astaxanthn pre-supplementation prior to CHI exposure in a mouse model.

The current study examined the effects of pre-treatment with Astaxanthin as a protective measure prior to TBI exposure. We have shown that the functional benefits attributed to Astaxanthin as post-injury treatment, previously demonstrated by Ji et al. in both motor and cognitive neuroprotection, as improvements in the NSS, and in the Y Maze and ORT tests, respectively (64), can be expanded to include prophylactic treatment, as demonstrated by the NSS results of the second stage gavage-treated ATX group, who achieved a significant improvement in ΔNSS over the OIL-treated group. ORT results of the gavage stage show a significant advantage for ATX over SAL, though not over OIL at 3 days postinjury. The differences observed in our work between the gavage-treated animal groups who did not receive additional treatment post-injury resulted only from pre-treatment. In the third stage of the study, we have shown that the more natural pellet form of administration used for supplementation conferred a shorter positive neuromotor effect in the NSS and additional cognitive benefits in the ORT and Y Maze tests late into recovery. Figure 5 summarizes the observed effects of Astaxanthin pre-treatment in the current study.

In humans, exposure to TBI induced a rise in brain tissue HSP72 protein levels measured in the initial hours at 10–24 h postinjury (89). An additional study displayed increased HSP72 conducted in the brain tissue from patients that had undergone surgical intervention after TBI (time point not mentioned) (90), suggesting involvement of the stress response, which was also the conclusion drawn by Lai et al. who analyzed cerebrospinal fluid samples of young patients suffering from TBI (91). Increased HSP72 serum levels have been shown to correlate with injury severity and a fatal outcome at 24 h−7 days post-injury in male patients (92). Indeed, upregulation of HSP70 gene expression was observed postmortem in human TBI patients by Michael et al. (93). The opposite was also reported in children from two studies, suggesting that high initial serum HSP72 levels upon arrival to the emergency room are predictive of survival post-TBI (94, 95). Here, we measured HSP72 protein levels in the impacted left cortical tissue to discover a gradual reduction trend, which became significant 8–24 h post-injury, before rising again, suggesting either increased protein utilization within the injured tissue or inhibited transcription in the initial hours post-injury. The underlying mechanisms for Astaxanthin-induced neuroprotection in TBI are complex and span beyond the scope of this work. Nevertheless, the non-significant trend observed toward increased left cortical levels of HSP72 and HSP27, though insufficient to determine HSP involvement at the site of injury, suggests potential influence for Astaxanthin on the HSP dynamics at the site of injury in the initial hours following TBI.

The neuroprotective effect associated with Astaxanthin has been demonstrated in various types of brain injury resilience: recently, in a rat model of subarachnoid hemorrhage, administration of Astaxanthin post-injury attenuated brain injury by improving mitochondrial function and neuronal survival (96), through involvement of sirtuin-1 and inhibition of the Toll-like receptor 4 signaling pathway (97). Similarly, Astaxanthin has been shown to reduce neuronal apoptosis when administered following spinal cord injury in rats (98). Notably, our group demonstrated similar neuroprotective effects, namely reduced apoptosis following CHI in mice in a cross-tolerance mechanism mediated by heat acclimation [Umschweif et al. (24)].

Astaxanthin's ability to penetrate the BBB, coupled with existing evidence of Astaxanthin's benefit in brain IRI models, implies a shared underlying mechanism that could potentially contribute to an improved response to the TBI model employed in this study of CHI. Neuronal preservation by Astaxanthin under stress conditions manifested by upregulation of, e.g., cAMP response element-binding protein (CREB) or brain-derived neurotrophic factor (BDNF) was also demonstrated *in vitro* (99). Glutamate-related excitotoxicity has been prevented by Astaxanthin pre-treatment in rats (100), possibly, through inhibiting endoplasmic reticulum stress caused by calcium influx (101).

The neuromotor manifestation of recovery demonstrated a significant and consistent advantage to ATX supplementation by gavage over OIL in the ΔNSS score (*p* < 0.003) throughout the recovery period. The pellet-fed group ATXpre_ATXpost displayed significant improvement of ΔNSS at 7 days post-CHI when compared to the post-treated SCpre_ATXpost group although for a shorter duration than the gavage form of administration of Astaxanthin (ATX).

A clear cognitive effect for Astaxanthin was shown in the ORT at 3 days for all pellet forms of Astaxanthin vs. SC in the change between the baseline and test stages of the test ΔER. At 30 days of recovery, advantage in both ORT and Y Maze results was apparent only for the pre-and post-treated group exposed to TBI over the sham-treated mice of the same group. This might mean that ongoing treatment during recovery is beneficial for longer lasting cognitive effects. This finding is in accordance with the fact that post-injury gavage-treated animals tested by Ji et al. demonstrated sustained improved effects in both the Y Maze and ORT tests throughout recovery (64).

The open field results suggest no significant change in anxiety.

In conclusion, we have demonstrated the benefit of Astaxanthin pre-treatment prior to exposure to CHI to both neuromotor and cognitive recovery, independent of treatment during recovery. Despite a more pronounced effect on neuromotor recovery conferred by the gavage form of administration, compared to free eating of pellets of Astaxanthin, a significant improvement in cognitive recovery—an important factor of function—was apparent in Astaxanthin administration by free eating. It is our impression that this form of administration is far less stressful for the animals, produces no change in appetite, as seen by the unaffected gain in body weight (Supplementary Table S2), and upon comparison to behavioral effects observed in the Y Maze and ORT tests, is superior to results obtained from the gavage form of administration (data not shown). Further analysis is required to elucidate the mechanism of action behind the different forms of administration and their influence on neuromotor and cognitive function in CHI; however, it was beyond the scope of the current investigation.

## Data Availability Statement

The raw data supporting the conclusions of this article will be made available by the authors, without undue reservation.

## Ethics Statement

The animal study was reviewed and approved by the authority for biological and biomedical models of The Hebrew University (Approval numbers: MD-13-13734-4 and MD-16-14842-4).

## Author Contributions

CF contributed to the conception and design of the study, conducted the experiments, analyzed the data, and wrote the manuscript. ES and MH contributed to the conception and design of the study and to data analysis and reviewed the manuscript. VT contributed to conducting the experiments and to data analysis and reviewed the manuscript. YH contributed to the conception of the study and reviewed the manuscript.

## Conflict of Interest

The authors declare that the research was conducted in the absence of any commercial or financial relationships that could be construed as a potential conflict of interest.

## Abbreviations

BBB: blood–brain barrier
BDNF: brain-derived neurotrophic factor
CHI: closed head injury
CREB: cAMP response element-binding protein
HSP: heat shock protein
HSPs: heat shock proteins
IRI: ischemia–reperfusion injury
NMDA: N-methyl-D-aspartate
NSS: neurological severity score
ΔNSS: change in neurological severity score
ORT: object recognition test
ROS: reactive oxygen species
TBI: traumatic brain injury
ΔER: change in exploration ratio
ΔNR: change in novelty ratio
ΔDI: change in discrimination index.
