# Supplementary material for: Cognitive Effects of Astaxanthin Pretreatment on Recovery From Traumatic Brain Injury
Source: Front Neurol. 2020 Oct 15;11:999. doi: 10.3389/fneur.2020.00999 (PMC7593578; doi:10.3389/fneur.2020.00999)
Supplement: Supplementary file 1 [file Data_Sheet_1.zip › Supplementary files/Supplementary Table 1.docx]

Supplementary table S1: Antibodies used for Western Blot protein detection

Table S1a: primary antibodies

| Primary antibody | | | | |
| --- | --- | --- | --- | --- |
| Protein detected | Manufacturer | cat. Number | Antibody origin | Dilution |
| Actin | Abcam, international | ab8227 | Rabbit | 1:5,000 |
| HSP27 | Santa cruz biotechnology, USA | (C-20): sc-1048 | Goat | 1:1,000 |
| HSP72 | StressMarq Biosciences Inc., Canada | SPC-103C/D | Rabbit | 1:10,000 |
| HSP90 | Abcam, international | AC88, ab13492 (monoclonal) | Mouse | 1:1,250 |
| HSF1 | Abcam, international | ab2923 | Rabbit | 1:1,000 |

Table S1b: secondary antibodies

| Secondary antibody (Horeseradish peroxidase conjugated IgG) | | | | |
| --- | --- | --- | --- | --- |
| Protein detected | Manufacturer | cat. Number | Antibody origin | Dilution |
| Rabbit IgG | Jackson ImmunoResearch laboratories Inc., USA | 711-035-152 | Donkey | 1:5,000 |
| Goat IgG | Jackson ImmunoResearch laboratories Inc., USA | 805-035-180 | Bovine | 1:10,000 |
| Mouse IgG | Jackson ImmunoResearch laboratories Inc., USA | 115-035-003 | Goat | 1:10,000 |
