# Supplementary figures and images for: Cognitive Effects of Astaxanthin Pretreatment on Recovery From Traumatic Brain Injury
Source: Front Neurol. 2020 Oct 15;11:999. doi: 10.3389/fneur.2020.00999 (PMC7593578; doi:10.3389/fneur.2020.00999)

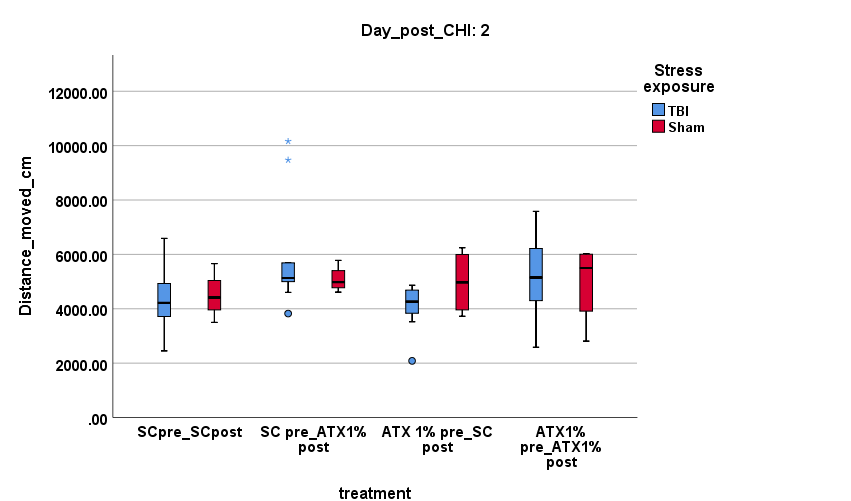

Supplement: Supplementary file 1 [file Data_Sheet_1.zip › Supplementary files/Figure S1a1 Distance moved in cm_day 2 post CHI.tif]

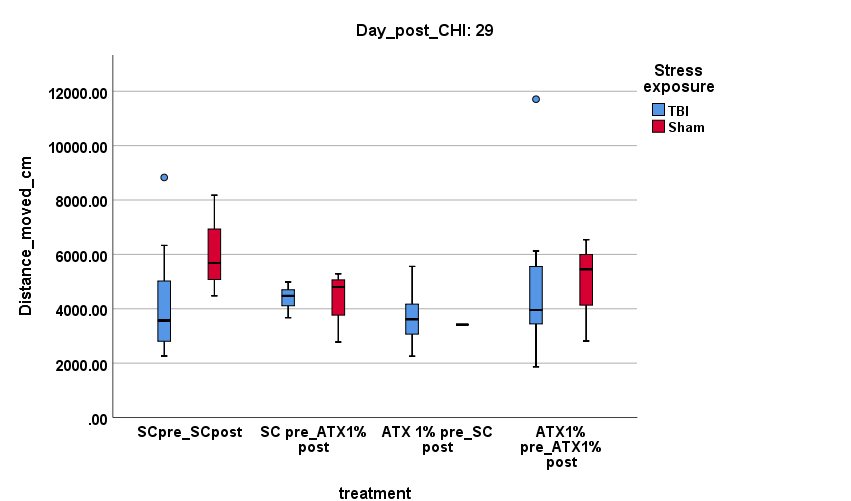

Supplement: Supplementary file 1 [file Data_Sheet_1.zip › Supplementary files/Figure S1a1 Distance moved in cm_day 29 post CHI.tif]

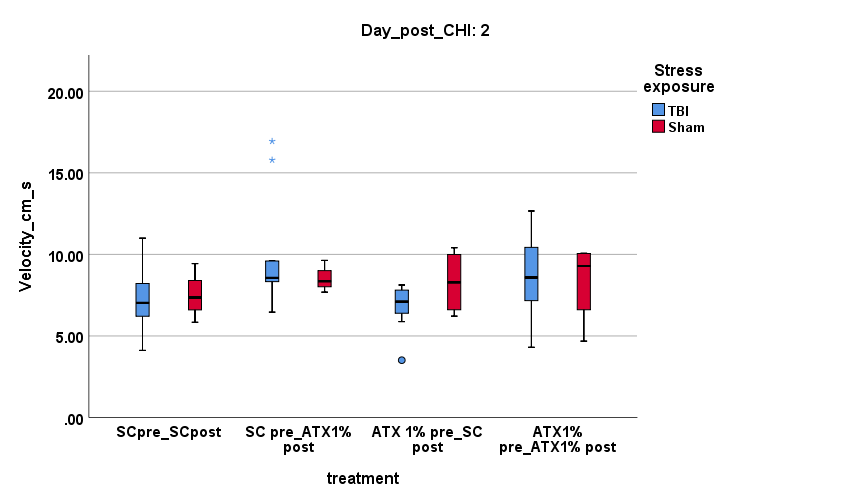

Supplement: Supplementary file 1 [file Data_Sheet_1.zip › Supplementary files/Figure S1b1 Velocity in cm X sec(-1) day 2 post CHI.png.tif]

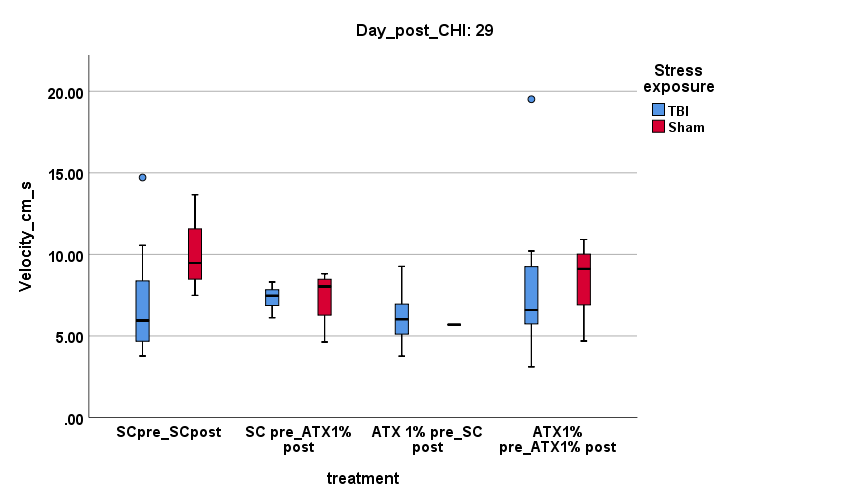

Supplement: Supplementary file 1 [file Data_Sheet_1.zip › Supplementary files/Figure S1b2 Velocity in cm X sec(-1) day 29 post CHI.tif]

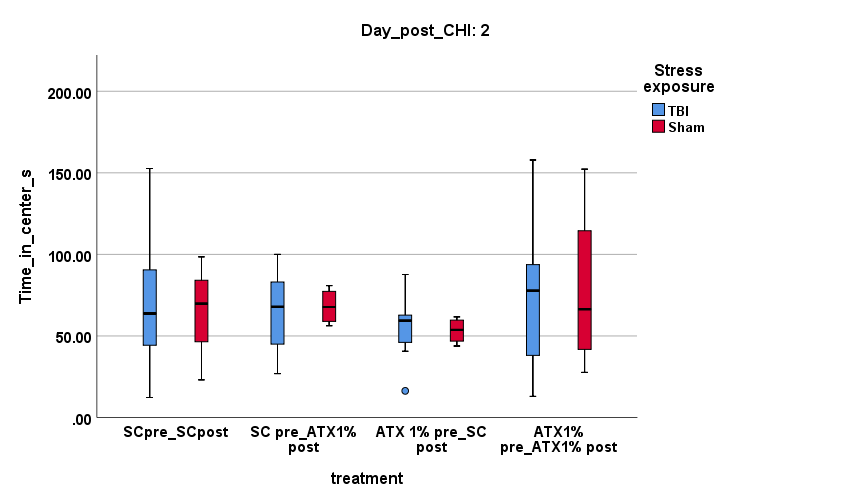

Supplement: Supplementary file 1 [file Data_Sheet_1.zip › Supplementary files/Figure S1c1 Time in center in sec._day 2 post CHI.tif]

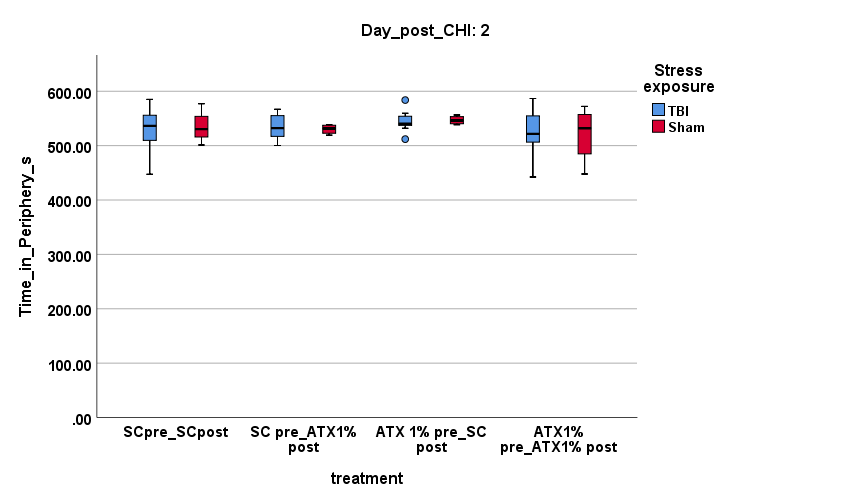

Supplement: Supplementary file 1 [file Data_Sheet_1.zip › Supplementary files/Figure S1d1 Time in periphery in sec._day 2 post CHI.tif]

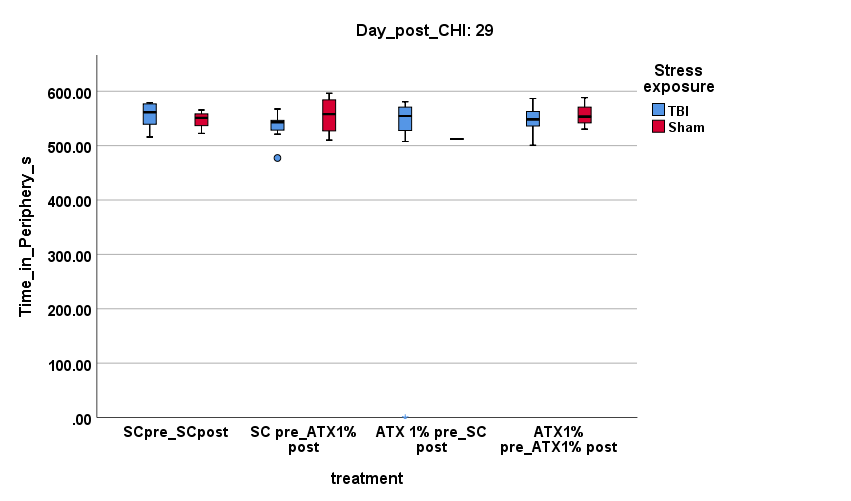

Supplement: Supplementary file 1 [file Data_Sheet_1.zip › Supplementary files/Figure S1d2 Time in periphery in sec._day 29 post CHI.tif]

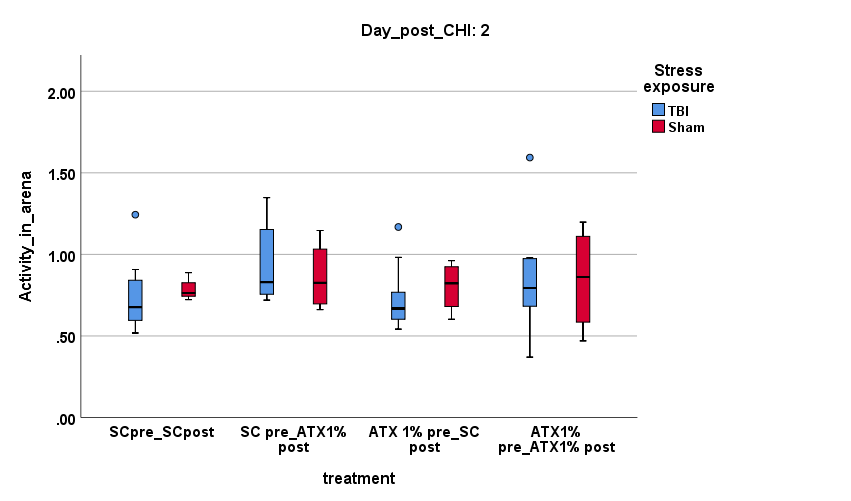

Supplement: Supplementary file 1 [file Data_Sheet_1.zip › Supplementary files/Figure S1e1 Activity in arena_day 2 post CHI.tif]

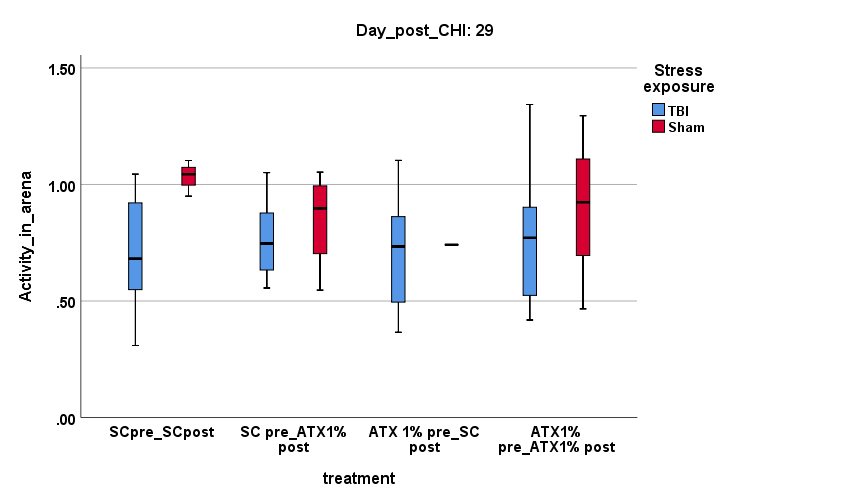

Supplement: Supplementary file 1 [file Data_Sheet_1.zip › Supplementary files/Figure S1e2 Activity in arena_day 29 post CHI.tif]

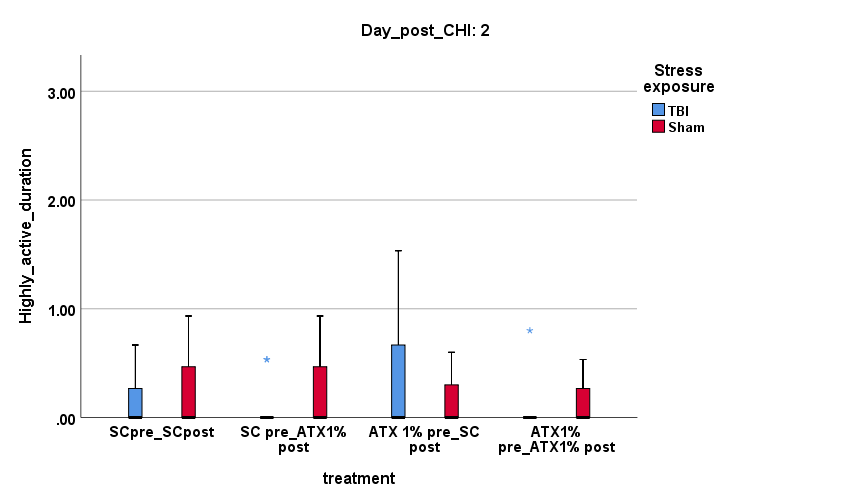

Supplement: Supplementary file 1 [file Data_Sheet_1.zip › Supplementary files/Figure S1f1 Highly active duration_day 2 post CHI.tif]

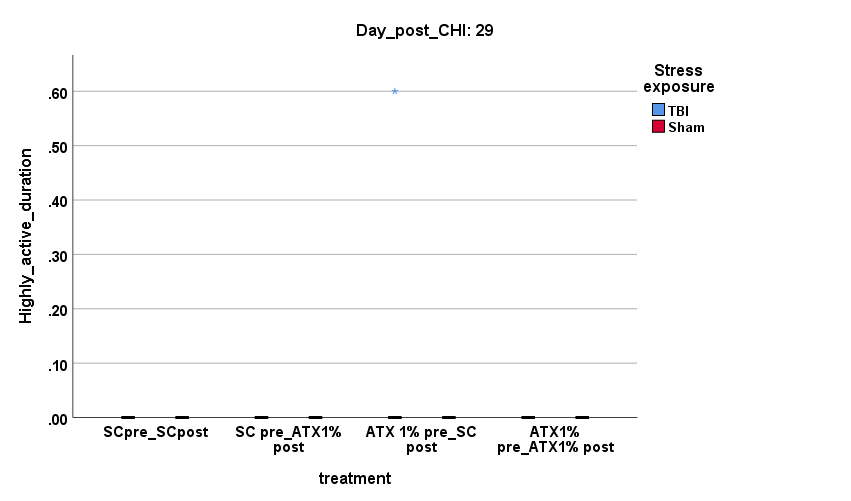

Supplement: Supplementary file 1 [file Data_Sheet_1.zip › Supplementary files/Figure S1f2 Highly active duration_day 29 post CHI.tif]

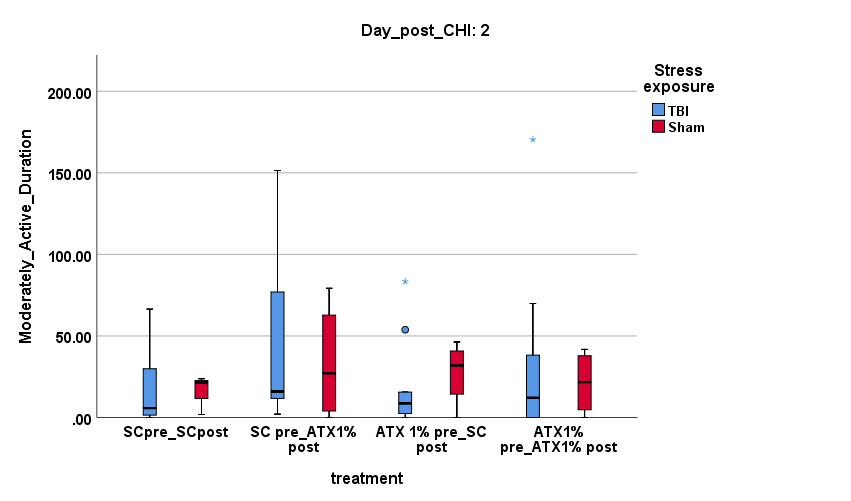

Supplement: Supplementary file 1 [file Data_Sheet_1.zip › Supplementary files/Figure S1g1 Moderately active duration_day 2 post CHI.tif]

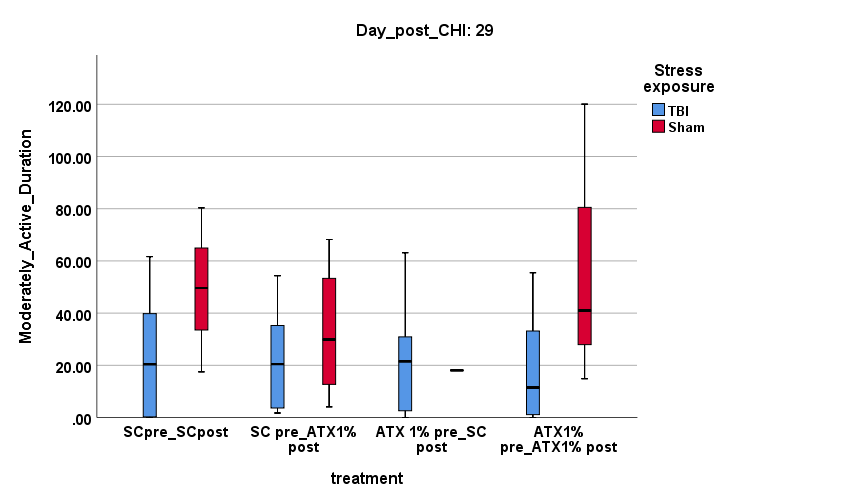

Supplement: Supplementary file 1 [file Data_Sheet_1.zip › Supplementary files/Figure S1g2 Moderately active duration_day 29 post CHI.tif]

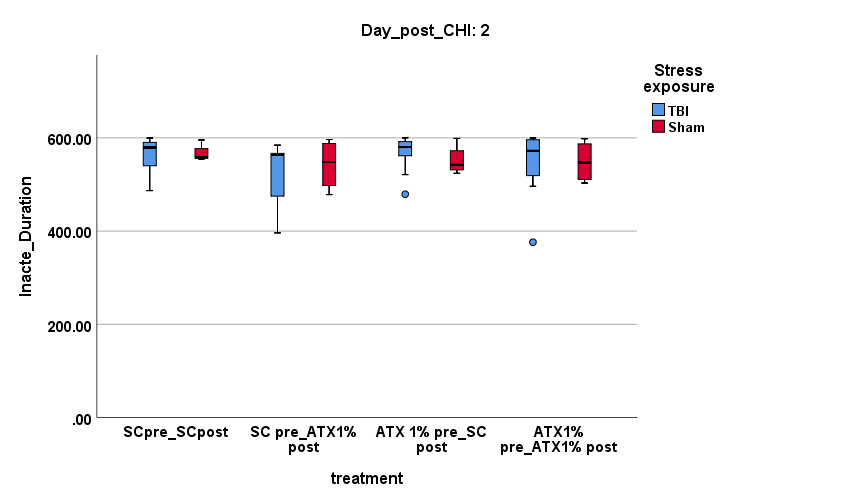

Supplement: Supplementary file 1 [file Data_Sheet_1.zip › Supplementary files/Figure S1h1 Inactive duration_day 2 post CHI.tif]

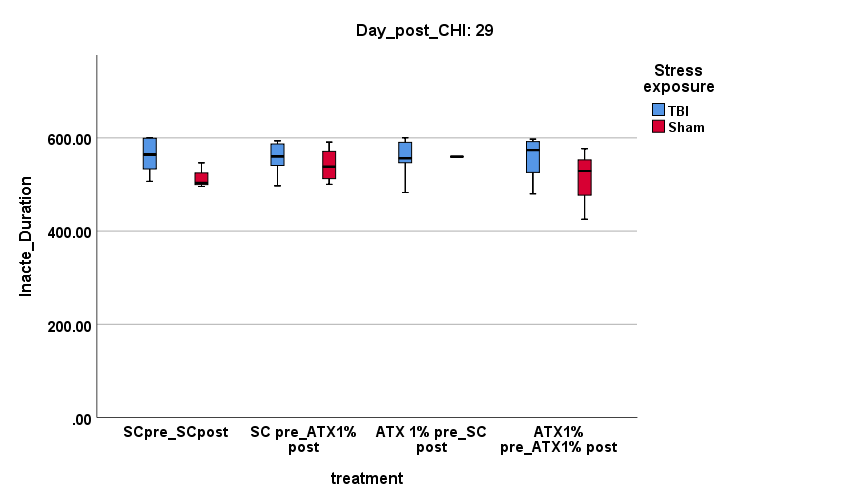

Supplement: Supplementary file 1 [file Data_Sheet_1.zip › Supplementary files/Figure S1h2 Inactive duration_day 29 post CHI.tif]

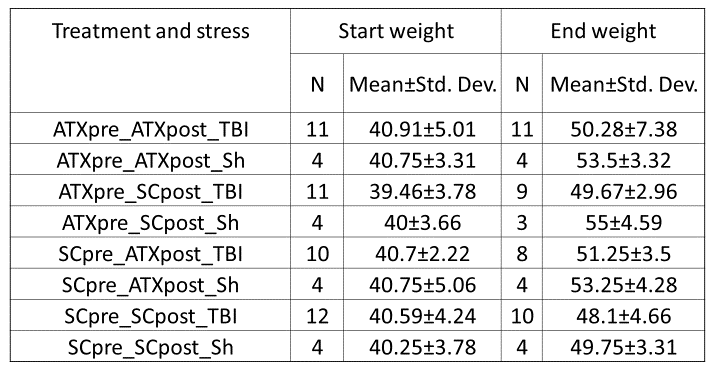

Supplement: Supplementary file 1 [file Data_Sheet_1.zip › Supplementary files/Supplementary Table 2.tiff]
